# First report of *Candida* (*Candidozyma) auris* infections in a human and a dolphin from the Dominican Republic: A One health perspective

**DOI:** 10.1016/j.mmcr.2025.100745

**Published:** 2025-10-18

**Authors:** Giuseppe A. Ferrara, Bram Spruijtenburg, Eelco F.J. Meijer, Jacques F. Meis, Andres Ceballos-Garzon, Diego H. Caceres

**Affiliations:** a Referencia Laboratorio Clínico, Santo Domingo, Dominican Republic; b Department of Medical Microbiology and Immunology, Canisius-Wilhelmina Hospital (CWZ)/Dicoon, Nijmegen, the Netherlands; c Radboudumc-CWZ Center of Expertise for Mycology, Nijmegen, the Netherlands; d Institute of Translational Research, Cologne Excellence Cluster on Cellular Stress Responses in Aging-Associated Diseases (CECAD), Excellence Center for Medical Mycology (ECMM), University of Cologne, Cologne, Germany; e Studies in Translational Microbiology and Emerging Diseases (MICROS) Research Group, School of Medicine and Health Sciences, Universidad del Rosario, Bogota, Colombia; f Immuno-Mycologics (IMMY), Norman, OK, USA

**Keywords:** Candida auris, Candidozyma auris, Antifungal resistance, Whole genome sequencing, Fluconazole, One health

## Abstract

This study documents the first reported cases of *Candida* (*Candidozyma)auris* in the Dominican Republic, isolated from both a critically ill human patient and a captive dolphin. Antifungal susceptibility testing from dolphin isolate showed elevated minimum inhibitory concentrations (MIC) of fluconazole but low MICs of other antifungals, including the novel drugs rezafungin, ibrexafungerp and manogepix. Whole genome sequencing (WGS) of the dolphin isolate allocated it to clade I, but surprisingly did not show any known mutations that confer azole resistance, suggesting a mechanism different from mutations in the genes studied. The concurrent emergence of *C. auris* in human and animal hosts underscores the importance of a One Health approach, emphasizing the interconnectedness of human, animal, and environmental health, and the need for strengthened surveillance and rigorous infection control measures across medical and veterinary settings.

## 1 Introduction

*Candida auris* (also known as *Candidozyma auris*) is an emerging multidrug-resistant yeast and a significant public health threat due to its ability to cause hospital outbreaks, its resistance to multiple antifungal drugs, and its misidentification [1]. *C. auris* has been detected on all inhabited continents, demonstrating its global presence [1,2]. The pathogen is known to persist in hospital environments, leading to healthcare-associated infections with high morbidity and mortality rates, particularly among critically ill patients [1,2].

Beyond human health, *C. auris* has recently been identified in various environmental and animal reservoirs, raising concerns about its zoonotic potential and the necessity of a One Health approach to study its epidemiology [[3], [4], [5], [6], [7], [8], [9]]. This report details the first case of *C. auris* infection in the Dominican Republic (Caribbean region) in a critically ill human patient and a case of colonization in a captive dolphin, emphasizing the role of advanced diagnostics for accurate identification and importance of enhanced surveillance.

## 2 Cases

### 2.1 Case 1 (human)

On September 1st, 2024, a blood culture was obtained from a 75-year-old male hospitalized at the intensive care unit (ICU). The specimen was processed using the BD BACTEC® Aerobic bottle system, with a positive alert on September 2nd, 2024. Gram staining showed yeast-like cells. Subcultures on blood agar and CHROMagar™ *Candida* were performed. Matrix-Assisted Laser Desorption Ionization–Time of Flight Mass Spectrometry (MALDI-TOF MS) (Bruker SIRIUS®) confirmed *C. auris* with a confidence score of 2.13 on September 3rd, 2024. However, Vitek® XL, automated microbial identification and antimicrobial susceptibility testing system, misidentified the isolate as *Meyerozyma guilliermondii* (formerly *Candida guilliermondii*)(90 %).

Antifungal susceptibility testing (AFST) results from Vitek® XL (AST-YST 08 card) and Etest showed: fluconazole >64 μg/mL, voriconazole 1 μg/mL, caspofungin 0.25 μg/mL, micafungin 0.12 μg/mL, and amphotericin B 0.5/mL.

The isolate from the human patient was not stored in the institution's biobank, which prevented confirmatory testing.

### 2.2 Case 2 (dolphin)

A pharyngeal specimen for routine animal care evaluation was collected from an 18-year-old male dolphin in captivity on November 15th, 2024, using a Transcult swab. The specimen was cultured on Sabouraud dextrose agar (SDA) with chloramphenicol and CHROMagar™ *Candida*, incubated at 26 ± 2 °C. Fungal growth was observed on November 17th, 2024, and subcultured on blood agar. On November 18th, 2024, MALDI-TOF MS confirmed *C. auris* with a confidence score of 2.05. As observed in the human isolate, the Vitek® XL incorrectly identified the organism as *M. guilliermondii*, with a confidence level of 92 %.

**Fig. 1:**
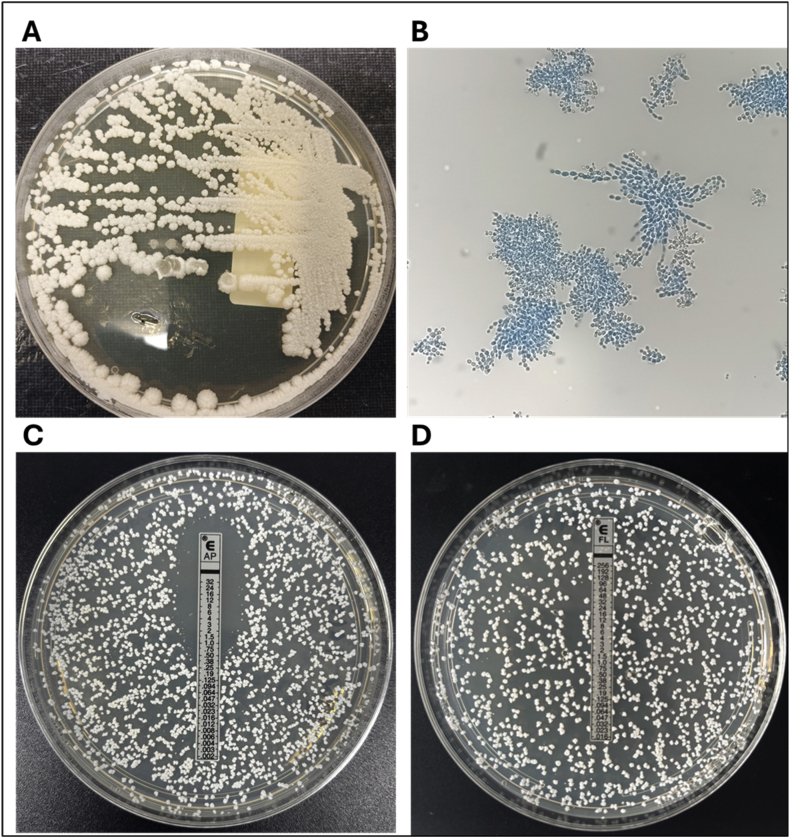
Morphological and antifungal susceptibility features of the *Candida auris* dolphin isolate. (A) Macro-morphology: Colonies grown on SDA at 25 °C displayed an off-white color, with a dull and dry appearance. The colony margins ranged from smooth to lobed, accompanied by a wrinkled texture. (B) Micro-morphology: Yeast cells appeared small, elongated to ovoid (2–4 μm), resembling long-grain rice and forming cells chains. (C) Amphotericin B (AP) and (D) Fluconazole (FL) Etest reading patterns, concentrations on the strip in micrograms per milliliter.

*Macro-morphology:* Colonies on SDA at 25 °C appeared off-white, dull, and dry, with margins ranging from smooth to lobed. The colonies exhibited a wrinkled surface texture (Fig. 1A). Additionally, the isolate demonstrated growth on SDA supplemented with 10 % NaCl at 42 °C.

*Micro-morphology:* Yeast cells were small, elongated to ovoid (2–4 μm), resembling long-grain rice and forming chains of cells (Fig. 1B).

AFST results obtained using Vitek® XL (AST-YST 08 card) showed MIC values of fluconazole 32 μg/mL, voriconazole 0.12 μg/mL, caspofungin 0.12 μg/mL, micafungin 0.06 μg/mL, and amphotericin B 0.5 μg/mL. Afterwards, the isolate was referred to external specialized laboratories for confirmatory testing, including broth microdilution (BMD) and whole-genome sequencing (WGS).

Further AFST was performed using CLSI BMD, M27 4th edition, for rezafungin (0.03 μg/mL), ibrexafungerp (0.06 μg/mL), and manogepix (<0.06 μg/mL), as well as Sensititre™ YeastOne™ (AST YO10 plate, TREK Diagnostic Systems, Cleveland, OH, USA), following manufacturers' instructions, for fluconazole (32 μg/mL), voriconazole (0.5 μg/mL), itraconazole (0.25 μg/mL), posaconazole (0.12 μg/mL), anidulafungin (0.015 μg/mL), caspofungin (0.015 μg/mL), micafungin (0.06 μg/mL), and amphotericin B (1 μg/mL). Additionally, Etest® (bioMérieux, Marcy-l’Étoile, France) results indicated fluconazole >256 μg/mL and amphotericin B 0.5 μg/mL (Fig. 1C and D).

**Fig. 2:**
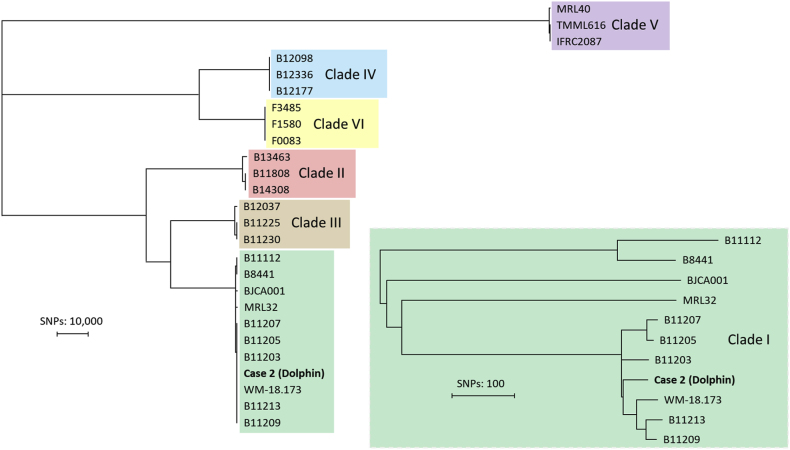
Whole genome sequencing (WGS) single nucleotide polymorphism (SNP) analysis of the dolphin isolate (Case 2) and control isolates spanning all described six clades.

Next, DNA was extracted using the MagNA Pure 96 instrument with the MagNA Pure DNA and Viral NA Small Volume Kit (Roche Diagnostics, Mannheim, Germany) according to manufacturer's instructions. For WGS, geonomic libraries were prepared and sequenced with the Illumina NovaSeq 6000 platform (Illumina, San Diego, CA, USA) with a 2- by 150-bp paired-end read mode. Reads were aligned to the B11220 (GCA_003013715.2) reference genome and variant calling was performed as previously described [10]. Control isolates spanning all six clades extracted from the NCBI database were included (Table 1). Raw read data generated in this study was submitted under BioProject ID. WGS SNP analysis allocated the isolate to clade I and was closely related to isolates from India (Fig. 2). The presence of resistance-associated genes *ERG11* (OL742093.1), *TAC1b* (OL742107.1), *MRR1* (OM287108.1), *FKS1* (OQ632644.1) and *ERG6* (OK564623.1) were examined in the current isolate and were visually inspected with IGV for missense mutations. This showed an absence of mutations that are associated with antifungal resistance. In contrast, all closely related isolates from India harbored either the Y132F or K143R mutation in *ERG11*. Additionally, no copy number variation (CNV) or large-scale deletions were found with YMAP v1.0 when compared to the reference genome B11220.

**Table 1:** Overview of control isolates included in the whole genome sequencing (WGS). analysis.

ID	Clade	*ERG11*	Country	SRA
B8441	I	WT	Pakistan	SRR10851769
B11112	I	WT	Pakistan	SRR3883473
B11203	I	Y132F	India	SRR14252434
B11205	I	K143R	India	SRR3883436
B11207	I	K143R	India	SRR3883439
B11209	I	Y132F	India	SRR3883441
B11213	I	Y132F	India	SRR3883444
WM-18.173	I	Y132F	India	SRR11485330
BJCA001	I	WT	China	SRR9316737
MRL32	I	WT	Iran	SRR25514773
B11808	II	WT	Japan	SRR10461263
B13463	II	WT	Canada	SRR10461159
B14308	II	WT	USA	SRR10461147
B11225	III	VF125AL	South Africa	SRR3883457
B11230	III	WT	South Africa	SRR3883463
B12037	III	WT	Canada	SRR10461253
B12098	IV	WT	Panama	SRR10461248
B12177	IV	Y132F	Venezuela	SRR10461201
B12336	IV	WT	Colombia	SRR7140028
IFRC2087	V	WT	Iran	SRR9007776
MRL40	V	I466L	Iran	SRR18325430
TMML616	V	WT	Iran	SRR18325431
F0083	VI	WT	Singapore	SRR25455197
F1580	VI	WT	Singapore	SRR25455198
F3485	VI	WT	Singapore	SRR25455199

## 3 Discussion

The detection of *C. auris* in both a critically ill human patient and a captive dolphin in the Dominican Republic raises significant concerns regarding the epidemiology, transmission, and resistance patterns of this emerging fungal pathogen.

In both cases, human and dolphin, the initial misidentification of *C. auris* as *M. guilliermondii* by the Vitek® XL system highlights the ongoing challenges in accurately diagnosing this multidrug-resistant yeast using conventional methods [11]. The correct identification by MALDI-TOF MS underscores the importance of advanced diagnostic tools in preventing misdiagnosis and ensuring appropriate patient management [12].

Based on the tentative resistance breakpoints established by the US Centers for Disease Control and Prevention (CDC), the strains should be classified as fluconazole-resistant (MIC ≥32 μg/mL) but susceptible to other antifungal agents [12]. The echinocandins, including the most recent addition rezafungin, along with the novel antifungals ibrexafungerp and manogepix, demonstrated potent *in vitro* activity with low MIC values. These findings corroborate previous reports of their strong antifungal efficacy, including activity against azole and echinocandin resistant strains [13,14]. Additionally, MICs values observed for common antifungals are consistent with global reports of clade I to which this isolate was assigned with WGS [15]. For clade I, the Y132F and K143R mutations in *ERG11* are found in nearly every isolate and these mutations are known to confer resistance [15]. Especially for azole resistance, the mechanism differs between clades and besides the *ERG11* gene, transcription factors such as *TAC1b* or *MRR1* can be involved [10,16]. Surprisingly, the isolate was closely related to other isolates that were fluconazole resistant as well, but no known mutations were found that could explain the elevated MIC. In Colombia, the same phenomenon was observed in seven isolates belonging to clade IV [17]. The absence of CNVs or deletions could suggest a novel azole resistance mechanism [18]. Genetic modification studies accompanied by genome-wide association analysis is needed for further investigation.

The identification of *C. auris* in a marine mammal is an unusual finding that raises questions about its zoonotic potential and environmental reservoirs. In this study, the human and animal cases occurred independently, and unfortunately, we did not have access to the human isolate for comparative analysis. However, previous studies have demonstrated the potential for *Saccharomycotina* yeasts to be transmitted across niches and species. For instance, in the case of C. albicans, the same genotype has been identified in a dolphin and a veterinary staff member responsible for its care [19]. Similarly, another study using six-gene MLST found that C. tropicalis isolates from humans, dolphins, and whales shared the same sequence types (ST232 and ST933) [20]. Adding to this concern are recently reported cases of *C. auris* in insects [21], snakes [22], dogs [4,23] and a domestic cat [24], where the yeast was repeatedly recovered from feces over a six-month period, indicating persistent intestinal colonization. It has been hypothesized that *C. auris* originated from marine environments, supported by the fact that another member of the species complex, *Candida haemulonii*, was first isolated in 1962 from an 10.13039/100004426Atlantic fish (*Haemulon sciurus*) off the coast of Florida, and seawater near Biscaine Bay and Lisbon, Portugal [25]. *C. auris* was detected in various environments, including animals, fruits, beach sand, seawater, and estuaries [3,[5], [6], [7],^26^,^27^]. Some studies identified it using genomic amplification alone. Only three studies from India determined the clade, all identifying clade I. Cetaceans in captivity are particularly vulnerable to fungal infections, largely due to stress-related factors associated with confinement and weakened immune responses. Notably, *Candida haemulonii* was identified as the predominant *Candida* species isolated from the environmental pools inhabited by captive bottlenose dolphins (*Tursiops truncatus*) [28]. The presence of *C. auris* in animal and environmental sources emphasizes the need for enhanced surveillance. The ability of *C. auris* to persist in the environment, coupled with its resistance to multiple antifungal agents, poses significant challenges for treatment and containment. Further molecular and genomic studies are essential to determine the genetic relationship between these isolates, assess potential transmission routes, and explore environmental reservoirs that may contribute to the spread of this pathogen.

The main limitation of this report was the lack of access to detailed clinical and epidemiological information for both cases, the human patient and the dolphin. This limitation restricted our ability to better contextualize the findings, assess potential risk factors, and explore possible routes of infection.

Overall, these findings underscore the importance of accurate diagnostic methodologies, continuous monitoring of antifungal resistance, and multidisciplinary collaboration between medical and veterinary fields to address the emerging threat posed by *C. auris*. The identification of *C. auris* in both human and animal hosts in the same geographical location highlights potential cross-species transmission and underscores the urgent need for stringent infection control measures. WGS SNP analysis highlights the spread of the well-established clade I although no known resistance mechanism was found. This case report aims to provide insights into the diagnostic challenges, antifungal resistance patterns, and broader implications for public and veterinary health, advocating for a multidisciplinary response to the emergence of this pathogen.

## CRediT authorship contribution statement

**Giuseppe A. Ferrara:** Writing – review & editing, Writing – original draft, Visualization, Validation, Supervision, Software, Resources, Project administration, Methodology, Investigation, Funding acquisition, Formal analysis, Data curation, Conceptualization. **Bram Spruijtenburg:** Writing – review & editing, Visualization, Validation, Software, Methodology, Investigation, Formal analysis, Data curation. **Eelco F.J. Meijer:** Writing – review & editing, Writing – original draft, Visualization, Validation, Supervision, Project administration, Methodology, Investigation, Funding acquisition, Formal analysis, Data curation. **Jacques F. Meis:** Writing – review & editing, Writing – original draft, Validation, Supervision, Methodology, Investigation, Formal analysis, Conceptualization. **Andres Ceballos-Garzon:** Writing – review & editing, Writing – original draft, Validation, Methodology, Investigation, Formal analysis, Data curation, Conceptualization. **Diego H. Caceres:** Writing – review & editing, Writing – original draft, Visualization, Validation, Supervision, Software, Resources, Project administration, Methodology, Investigation, Funding acquisition, Formal analysis, Data curation, Conceptualization.

## Conflict of interest

Since December 2021, Diego H. Caceres has been an IMMY employee, serving as the Latin America Account Manager. Eelco F.J. Meijer received research grants from 10.13039/100030679Mundipharma and Scynexis, is in the scientific advisory board for 10.13039/100004319Pfizer and has received speaker fees from 10.13039/100005564Gilead Sciences. All other authors declare no conflict of interest.

## References

[1] Chakrabarti A., Sood P. (2021). On the emergence, spread and resistance of candida auris: host, pathogen and environmental tipping points. J. Med. Microbiol..

[2] Meis J.F., Chowdhary A. (2018). Candida auris: a global fungal public health threat. Lancet Infect. Dis..

[3] Nwaubani D.A., Baral R., Solomon T., Idris O., Sherchan S.P. (2025). Wastewater surveillance of Candida auris in Baltimore. Int. J. Hyg Environ. Health.

[4] White T.C., Esquivel B.D., Rouse Salcido E.M., Schweiker A.M., Dos Santos A.R., Gade L. (2024). Candida auris detected in the oral cavity of a dog in Kansas. mBio.

[5] Escandón P. (2023). Novel environmental niches for Candida auris: isolation from a coastal habitat in Colombia. J Fungi (Basel).

[6] Yadav A., Jain K., Wang Y., Pawar K., Kaur H., Sharma K.K. (2022). Candida auris on apples: diversity and clinical significance. mBio.

[7] Arora P., Singh P., Wang Y., Yadav A., Pawar K., Singh A. (2021). Environmental isolation of Candida auris from the coastal wetlands of andaman Islands, India. mBio.

[8] Sexton D.J., Kordalewska M., Bentz M.L., Welsh R.M., Perlin D.S., Litvintseva A.P. (2018). Direct detection of emergent fungal pathogen Candida auris in clinical skin swabs by SYBR green-based quantitative PCR assay. J. Clin. Microbiol..

[9] Welsh R.M., Bentz M.L., Shams A., Houston H., Lyons A., Rose L.J. (2017). Survival, persistence, and isolation of the emerging multidrug-resistant pathogenic yeast Candida auris on a plastic healthcare surface. J. Clin. Microbiol..

[10] Thakur S., Spruijtenburg B., Shaw D., Abhishek, de Groot T., Meijer E.F.J. (2025). Whole genome sequence analysis of terbinafine resistant and susceptible Trichophyton isolates from human and animal origin. Mycopathologia.

[11] Ambaraghassi G., Dufresne P.J., Dufresne S.F., Vallieres E., Munoz J.F., Cuomo C.A. (2019). Identification of Candida auris using the updated 8.01 VITEK(R)2 yeast identification system: a multi-laboratory evaluation study. J. Clin. Microbiol..

[12] CDC (2025). *Candida auris* (*C. auris*). https://www.cdc.gov/candida-auris/index.html.

[13] Hoenigl M., Sprute R., Egger M., Arastehfar A., Cornely O.A., Krause R. (2021). The antifungal pipeline: fosmanogepix, ibrexafungerp, olorofim, opelconazole, and rezafungin. Drugs.

[14] Ceballos-Garzon A., Lebrat J., Holzapfel M., Josa D.F., Welsch J., Mercer D. (2025). Antibiofilm activity of manogepix, ibrexafungerp, amphotericin B, rezafungin, and caspofungin against Candida spp. biofilms of reference and clinical strains. Antimicrob. Agents Chemother..

[15] Chowdhary A., Prakash A., Sharma C., Kordalewska M., Kumar A., Sarma S. (2018). A multicentre study of antifungal susceptibility patterns among 350 Candida auris isolates (2009-17) in India: role of the ERG11 and FKS1 genes in azole and echinocandin resistance. J. Antimicrob. Chemother..

[16] Ceballos-Garzon A, Peñuela A, Valderrama-Beltrán S, Vargas-Casanova Y, Ariza B, Parra-Giraldo CM (2023 Mar 21). Emergence and circulation of azole-resistant C. albicans, C. auris and C. parapsilosis bloodstream isolates carrying Y132F, K143R or T220L Erg11p substitutions in Colombia. Front Cell Infect Microbiol..

[17] Alvarado Casas M.L., Triana Díaz J.A., Montilla-Escudero E., Prada Cardozo D.A., Escandón Hernández P. (2025). Genomic epidemiology of Colombian resistant Candida auris isolates. Antimicrob. Agents Chemother..

[18] Su Y., Li Y., Yi Q., Xu Y., Sun T. (2025). Insight into the mechanisms and clinical relevance of antifungal heteroresistance. J Fungi (Basel).

[19] Takahashi H., Ueda K., Itano E.N., Yanagisawa M., Murata Y., Murata M. (2010). Candida albicans and C. tropicalis isolates from the expired breathes of captive dolphins and their environments in an aquarium. Vet. Med. Int..

[20] Khalifa HO, Watanabe A, Kamei K. Azole and echinocandin resistance mechanisms and genotyping of Candida tropicalis in Japan: cross-boundary dissemination and animal-human transmission of C. tropicalis infection. Clin. Microbiol. Infect.. 28. p. 302 e5- e8 (2022). 10.1016/j.cmi.2021.10.004 34687855

[21] Ogundeji A., Bello-Akinosho M., Swart V., Featherston J., Cason E.D., Bolsenbroek A. (2025). Brown locusts, Locustana pardalina, host fluconazole-resistant Candidozyma (Candida) auris, closely related to Clade III clinical strains. Med. Mycol..

[22] Cafarchia C., Mendoza-Roldan J.A., Rhimi W., I C.I.U., Miglianti M., Beugnet F. (2024). Candida auris from the Egyptian cobra: role of snakes as potential reservoirs. Med. Mycol..

[23] Yadav A., Wang Y., Jain K., Panwar V.A.R., Kaur H., Kasana V. (2023). Candida auris in dog ears. J Fungi (Basel).

[24] Grassi A., Rigamonti S., Danesi P., Olivieri E., Sgubin S., Prati P. (2025). First report of Candidozyma auris (Candida auris) in a cat: a case of persistent shedding. Medical Mycology Case Reports.

[25] van Uden, Kolipinski M.C. (1962). Torulopsis haemulonii Nov. spec., a yeast from the Atlantic Ocean. Antonie Leeuwenhoek.

[26] Spruijtenburg B., Nobrega de Almeida Júnior J., Ribeiro F.C., Kemmerich K.K., Baeta K., Meijer E.F.J. (2024). Multicenter Candida auris outbreak caused by azole-susceptible clade IV in Pernambuco, Brazil. Mycoses.

[27] Babler K., Sharkey M., Arenas S., Amirali A., Beaver C., Comerford S. (2023). Detection of the clinically persistent, pathogenic yeast spp. Candida auris from hospital and municipal wastewater in Miami-Dade County, Florida. Sci. Total Environ..

[28] Buck J.D. (1980). Occurrence of human-associated yeasts in the feces and pool waters of captive bottlenosed dolphins (Tursiops truncatus). J. Wildl. Dis..

